# Susceptibility of *Campylobacter* Strains to Selected Natural Products and Frontline Antibiotics

**DOI:** 10.3390/antibiotics9110790

**Published:** 2020-11-09

**Authors:** Noel Gahamanyi, Dae-Geun Song, Kwang Hyun Cha, Kye-Yoon Yoon, Leonard E.G. Mboera, Mecky I. Matee, Dieudonné Mutangana, Raghavendra G. Amachawadi, Erick V.G. Komba, Cheol-Ho Pan

**Affiliations:** 1 Natural Product Informatics Research Center, KIST Gangneung Institute of Natural Products, Gangneung 25451, Korea; noel.gahamanyi@kist.re.kr (N.G.); dsong82@kist.re.kr (D.-G.S.); chakh79@kist.re.kr (K.H.C.); yoonky@kist.re.kr (K.-Y.Y.); 2 SACIDS Foundation for One Health, College of Veterinary Medicine and Biomedical Sciences, Sokoine University of Agriculture, Chuo Kikuu, Morogoro P.O. Box 3015, Tanzania; leonard.mboera@sacids.org (L.E.G.M.); ekomba@sua.ac.tz (E.V.G.K.); 3 School of Medicine, Muhimbili University of Health and Allied Sciences, Dar es Salaam P.O. Box 65001, Tanzania; mecky.matee@sacids.org; 4 College of Science and Technology, University of Rwanda, Kigali P.O. Box 3900, Rwanda; d.mutangana@ur.ac.rw; 5 Department of Clinical Sciences, College of Veterinary Medicine, Kansas State University, Manhattan, KS 66506-5606, USA; agraghav@vet.k-state.edu; 6 Division of Bio-Medical Science and Technology, KIST School, Korea University of Science and Technology, Seoul 02792, Korea

**Keywords:** antibiotics, *Campylobacter*, plant extracts, essential oils, phytochemicals, resistance

## Abstract

*Campylobacter* species have developed resistance to existing antibiotics. The development of alternative therapies is, therefore, a necessity. This study evaluates the susceptibility of *Campylobacter* strains to selected natural products (NPs) and frontline antibiotics. Two *C. jejuni* strains (ATCC^®^ 33560^TM^ and MT947450) and two *C. coli* strains (ATCC^®^ 33559^TM^ and MT947451) were used. The antimicrobial potential of the NPs, including plant extracts, essential oils, and pure phytochemicals, was evaluated by broth microdilution. The growth was measured by spectrophotometry and iodonitrotetrazolium chloride. Antibiotic resistance genes (*tet*(O) and *gyr*A) were characterized at the molecular level. The minimum inhibitory concentrations (MICs) and the minimum bactericidal concentrations (MBCs) ranged from 25 to 1600 µg/mL. Cinnamon oil, (E)-Cinnamaldehyde, clove oil, eugenol, and baicalein had the lowest MIC and MBC values (25–100 µg/mL). MT947450 and MT947451 were sensitive to erythromycin and gentamicin but resistant to quinolones and tetracycline. Mutations in *gyr*A and *tet*(O) genes from resistant strains were confirmed by sequencing. The findings show that NPs are effective against drug-sensitive and drug-resistant *Campylobacter* strains. The resistance to antibiotics was confirmed at phenotypic and genotypic levels. This merits further studies to decipher the action mechanisms and synergistic activities of NPs.

## 1. Introduction

*Campylobacter* species, mainly *C. jejuni* and *C. coli*, are among the major pathogens causing human gastroenteritis [1]. Campylobacteriosis is of public health concern in low-, middle-, and high-income countries [2]. Biofilm formation in *Campylobacter* contributes to its resistance to environmental stress and antibiotics [3]. Human infections with *Campylobacter* species occur via the ingestion of contaminated animal products or water [4,5,6].

There have been increased reports about high-level resistance to frontline and alternative antimicrobials, including macrolides, fluoroquinolones, aminoglycosides, and tetracyclines, among *Campylobacter* strains [7,8]. Increased antimicrobial resistance (AMR) among pathogens has been associated with many factors, including the unrestricted use of antimicrobials in various fields [9,10]. The main mechanisms of AMR include mutations in specific genes and acquiring efflux pumps [11]. For instance, the main resistance mechanism to ciprofloxacin is through target mutation in the DNA *gyr*A gene, along with the *CmeABC* efflux pump [4,12]; the majority (75–90%) of *Campylobacter* isolates worldwide have developed resistance to this important category of antibiotics [13]. The resistance to tetracycline is known to be either on a plasmid or bacterial chromosome [14,15,16]. It is estimated that by the year 2050, if no adequate actions are taken, the annual death rate due to AMR would reach 10 million people worldwide and cost USD 100 trillion [17]. Poultry has been recognized as the primary reservoir of *Campylobacter* strains that are resistant to fluoroquinolones associated with human diseases [18]. The progressive end of the traditional antimicrobial drug era as a result of the increasing number of AMR pathogens requires the development of new approaches to deal with AMR pathogens [11,19].

To improve the current trend, natural products (NPs) are good candidates in food preservation and/or drug development due to their rich composition [20,21]. Herbal medicines, generally recognized as safe, are more widely used and more affordable than synthetic ones [22,23]. NPs are also known to work in synergy with existing drugs to combat AMR pathogens [24]. Although the literature on the anti-*Campylobacter* activity of natural products is scanty, *Cinnamomum* cassia (L.) J.Presl is a known traditional Chinese medicine for treating various diseases, while *Scutellaria baicalensis* Georgi is effective against *Helicobacter pylori* [25,26], which is phylogenetically closely related to *Campylobacter* [2]. Studies have shown that cinnamon oil works well against *Campylobacter* species and several other pathogens [27,28,29]. *Mentha canadensis* L. proved to inhibit both *C. jejuni* and *H. pylori* [30,31] and it is also effective in the treatment of dysentery [31]. *Meehania urticifolia* (Miq.) Makino is known for its phenolic compounds but its antimicrobial activity is still poorly reported [32]. Clove oil and its major phytochemical eugenol are known for their antimicrobial and virulence-modulating activities against *Campylobacter* species [28,33]. Emodin has been found to inhibit *Pseudomonas aeruginosa* [34], while kuraridin had activity against different pathogenic bacteria [35] and reoviruses [36]. Cinnamaldehyde has been reported to possess antimicrobial properties against various pathogens [37]. Therefore, NPs could become potential sources for ensuring the safety of food items during this period when resistance to antimicrobials and tolerance to methods used in food industries are escalating [38,39]. 

It has been recognized that medicinal plants are equipped with bioactive compounds used for prophylaxis and therapeutic purposes [40]. It is estimated that 87% of populations from low- and middle-income countries rely on medicinal plants for their healthcare [41,42,43]. Several millions of NPs exist but only a small number of them have been explored for anti-*Campylobacter* activities. Considering that *Campylobacter* is a public health concern and one of the pathogens on the World Health Organization (WHO) list for which drug development is an emergency [42,43], it is imperative to explore possible alternative solutions through the use of NPs that can be candidates for drug development. With this background, the present study evaluates the susceptibility of *Campylobacter* strains to selected NPs and frontline antimicrobials. 

## 2. Results

**Figure 1:**
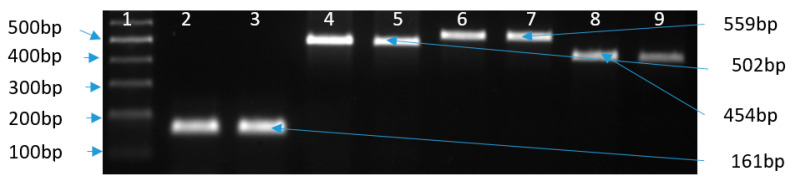
Agarose gel image showing bands of *C. jejuni*, *C. coli*, *tet*(O), and *gyr*A, where 1: marker; 2: CJ-RS; 3: CJ-CI; 4: CC-RS; 5: CC-CI; 6–7: *tet*(O) gene (559 bp), and 8–9: *gyr*A gene (454 bp) from antibiotic-resistant strains (CJ–CI and CC–CI).

Each *Campylobacter* strain was confirmed to species level based on culture, PCR products (Figure 1), and sequencing. A basic local alignment search tool (BLAST) analysis showed a 99% similarity between *C. coli* from chicken (CC–CI) and *C. coli* YH502 (CP018900.1) isolated from retail chicken. The BLAST also showed a 100% similarity between *C. jejuni* from chicken (CJ–CI) and *C. jejuni* (CP047481.1) isolated from patients with gastrointestinal disease in Chile. The detection rates and species distribution related to fecal samples collected from the poultry farm are not presented in this manuscript.

The five plant extracts (Table 1), along with essential oils (EOs), pure phytochemicals, and antibiotics, were tested against four *Campylobacter* strains. The concentrations used ranged from 25–6400 µg/mL for plant extracts, 6.25–1600 µg/mL for EOs and phytochemicals, and 0.06–512 µg/mL for antibiotics.

For *C. jejuni* strains, the MIC values for extracts were from 200–800 µg/mL, with *C. cassia* being the most active against all the four strains (MIC: 200 µg/mL). The MIC value for the other extracts was 400 µg/mL, except for *Mentha canadensis* L. and *Salvia plebeia* R.Br. against CJ–CI, which had higher values (MIC: 800 µg/mL). The MIC values for EOs, pure phytochemicals, and ERY were the same for both CJ–RS and CJ–CI. In contrast, CJ–CI showed resistance to ciprofloxacin, nalidixic acid, and tetracycline while CJ–RS was sensitive to all antimicrobials (Table 2).

For the EOs, cinnamon oil, and its phytochemical (E)- Cinnamaldehyde had the lowest MIC of 25–50 µg/mL against all tested strains. For baicalein, a phytochemical from *S. baicalensis*, the MIC values were 32 and 64 µg/mL for *C. jejuni* and *C. coli*, respectively. For kuraridin, the MIC value was 48 µg/mL for all the four strains, while for emodin, the MIC values were 50 µg/mL for *C. jejuni* and 200 µg/mL for *C. coli*. 

For *C. coli* strains, the MIC value for *C. cassia* was 200 µg/mL, while the MIC value for the remaining extracts was 400 µg/mL. The MIC values for EOs and pure phytochemicals were the same for both *C. coli* strains. However, for antibiotics, CC–CI was resistant to CIP, NAL, and TET, while CC–RS was sensitive to all used antimicrobials. 

In general, the strains isolated from chicken showed sensitivity to gentamicin and erythromycin, but they were resistant to quinolones and tetracycline (Table 2).

**Figure 2:**
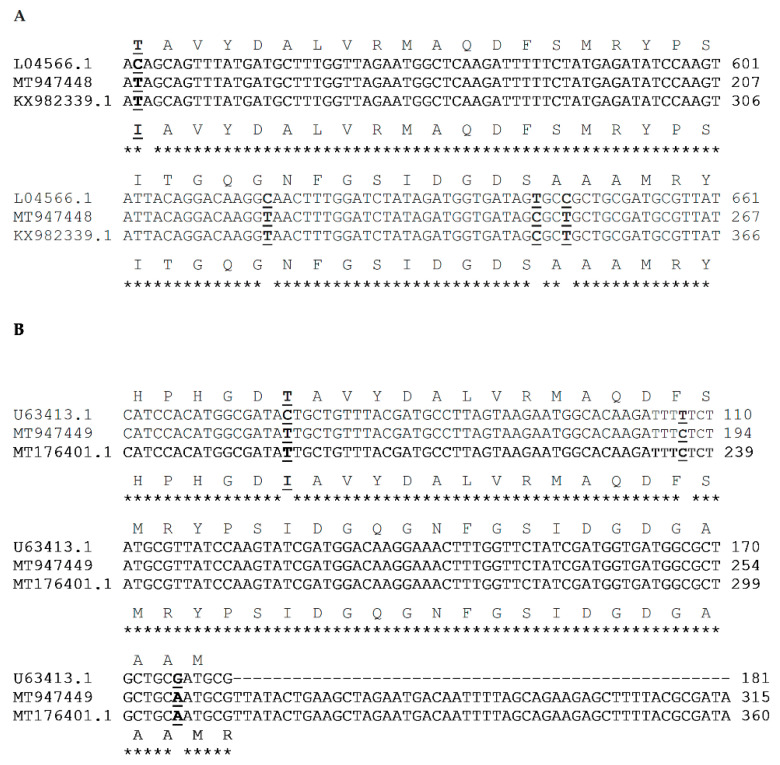
Mutations in *gyr*A sequences of *C. jejuni* (**A**) and *C. coli* (**B**). The mutation (Thr86Ile) is caused by the change from ACA to ATA (*C. jejuni*) and ACT to ATT (*C. coli*). Silent mutations in *gyr*A are also depicted. Mutations are bolded and underlined. L04566.1 and U63413.1 are standard strains (without mutation), while KX982339.1 and MT176401.1 are resistant strains. MT947448 and MT947449 are chicken isolates of this study.

The MBC values for all the strains ranged from 25–1600 µg/mL, with (E)-Cinnamaldehyde and cinnamon oil showing the lowest values (25–100 µg/mL). The MBC values for plant extracts varied between 400 and 1600 µg/mL, with *C. cassia* showing the lowest MBC of 400 µg/mL. The MBC values for ciprofloxacin and nalidixic acid varied between 64 and 256 µg/mL, while it varied between 128 and 512 µg/mL for tetracycline for the chicken isolates (Table 2). The PCR results show that chicken isolates possess *gyr*A and *tet*(O) genes, which confirm the phenotypic results from MIC determination (Figure 1). However, *cme*B was absent in both *Campylobacter* isolated from chicken by PCR. After sequencing PCR products, chicken isolates exhibited mutations for *gyr*A and *tet*(O). The Thr86Ile point mutation for *C. jejuni* and *C. coli*, associated with resistance to quinolones (CIP and NAL), was confirmed by the sequencing of PCR products (Figure 2).

The Genbank accession numbers registered for DNA *gy*rA sequences of *C. jejuni* and *C. coli* in this study are MT947448 and MT947449, respectively. Furthermore, MT947448 and MT947449 exhibited two silent mutations each (AGT to AGC for Ser119Ser and GCC to GCT for Ala120Ala in *C. jejuni*; TTT to TTC for Phe99Phe and GCG to GCA for Ala122Ala in *C. coli*). The sequences for *gyr*A genes showed similarity to known sequences from GenBank (Figure 2).

The resistance to tetracycline was confirmed to be plasmid-mediated as the *tet*(O) gene of *C. jejuni* (MT967269) and *C. coli* (MT967270) exhibited 100% similarity with *tet* (O)-resistance genes of *C. jejuni* and *C. coli* sequences in Genbank (data not shown).

## 3. Discussion

The objective of this study is to evaluate the susceptibility of *Campylobacter* strains to various NPs and frontline antibiotics. Cinnamon extract, oil, and trans (E)-Cinnamaldehyde had the lowest anti-*Campylobacter* activities, ranging from 25 to 200 µg/mL, which concurs with previous results where the range was from 46.8–600 µg/mL [28,44,45]. However, the MIC for cinnamon oil was lower than the 1000 µg/mL reported against *Campylobacter* strains in Egypt [28]. Clove oil and its major compound eugenol had MICs varying from 50–100 µg/mL, which are higher than the previously reported value of 20 µg/mL for clove oil [33] but lower than the 500 µg/mL reported for eugenol [28]. Other studies have also reported the strong anti-*Campylobacter* potential of cinnamon and clove oil [45,46]. Essential oils are given to broilers to control *Campylobacter* [46]. The difference in MIC values could be associated with the method used as some researchers used an agar-based method instead of the recommended broth microdilution [28]. Other probable reasons could be the location and extraction procedures [47] or the presence of biofilm, virulence, and antibiotic-resistance genes [3].

Except for cinnamon, other extracts had MIC values varying between 400 and 800 µg/mL. The susceptibility of screened extracts was found to be moderate to weak according to the classification of Kuete, where the activity is considered as significant (MIC < 100 μg/mL), moderate (100 < MIC < 625 μg/mL), and weak (MIC > 625 μg/mL) [48]. There is a dearth of information on the biological activity of *M. urticifolia*. However, it is expected to have antimicrobial activities attributed to phenolic compounds and hyaluronidase inhibitory phenylpropanoids [32,49,50]. *Scutellaria baicalensis* Georgi is used in the treatment of *H. pylori* infections, and it is advocated to be a source of new drugs against *H. pylori, which is* closely related to *Campylobacter* [26,51]. *Scutellaria baicalensis* Georgi has also been reported to inhibit *Staphylococcus aureus* [52]. Baicalein, a major compound from *S. baicalensis* Georgi, had a MIC of 32–64 µg/mL, which concurs with the previous report on *S. aureus* [53]. *Mentha canadensis* L., known as an antidiarrheic and antidysentery plant [31], has been reported to inhibit *H. pylori* and *C. jejuni* [30,31]. It possesses monoterpenes, mainly menthol, which increases membrane permeability, leading to the loss of intracellular contents [31]. The antimicrobial activities of *S. plebeia* on different pathogens have been extensively reported [54].

The MIC of Kuradin against all isolates was 48 µg/mL which is more or less similar to the value of 50 µg/mL reported for *S. aureus* [55], but higher than a value of 20 µg/mL previously reported for different bacteria [35]. Kuraridin, from *Sophora flavescens*, has been previously reported as a potential antimicrobial compound [35,36,56]. The MIC of emodin against *C. jejuni* was 50 µg/mL, which is slightly lower than the 70–90 µg/mL previously reported for *P. aeruginosa* and *S. aureus* [34]. However, the MIC of 200 µg/mL against *C. coli* was higher than the reported values by Basu et al. [34]. The literature on both kuraridin and emodin is scanty, and there are no previous findings against *Campylobacter* species. Further studies on *Campylobacter* strains from different sources are needed to confirm the effectiveness of both kuraridin and emodin.

The chicken isolates exhibited resistance to quinolones (CIP and NAL) and tetracycline. These results support previous reports of increased resistance to fluoroquinolones in *C. jejuni* strains from various sources, including chickens [57]. *Campylobacter* strains are becoming more resistant to drugs of choice, and this has been associated with the irrational use of various antibiotics in animal husbandry [58], mainly poultry [7,8]. Apart from the point mutation in *gyr*A, increased resistance to quinolones has been associated with the broad use of fluoroquinolones in the human population and veterinary medicine [59]. Furthermore, ciprofloxacin is used in treating diarrhea cases of unknown etiology, and once acquired, resistance to fluoroquinolones can be maintained in populations even after being banned in animal production [18]. In South Korea, the use of fluoroquinolones in veterinary medicine was banned in July 2020 [60,61]. The mutation in *gyr*A (Thr86Ile) confers resistance to ciprofloxacin and nalidixic acid [62]. However, a different mutation (Thr86Ala) in *gyr*A has been associated with resistance to nalidixic acid alone [63]. The Thr86Ile mutation was found in *Campylobacter* species isolated from chicken (this study), which is in agreement with the broth microdilution and PCR results. The same mutation has been associated with high-level resistance to quinolones [63,64].

The sequence of the *tet*(O) gene (MT967269 and MT967270) was similar to other *tet*(O) genes from the Genbank strains (81-176, NG_048260.1, CP044175.1). The high-level resistance to tetracycline is common in *Campylobacter* strains isolated from humans and broilers [6], and it has been attributed to the *tet*(O) gene found either on plasmids or bacterial chromosome [15,16]. The used NPs inhibited all the strains, including those resistant to tested quinolones and tetracycline. 

The ultimate goal of screening for antimicrobial activities from plant-derived products is to avail ourselves of products with antipathogenic and anti-inflammatory potencies that can be used in either prevention or treatment of campylobacteriosis [65]. However, in vivo studies for the anti-*Campylobacter* activities of NPs are limited, possibly due to a lack of suitable infection models [66]. For instance, Hlashwayo et al. [42] recently reported that not even a single in-vivo study had been published in sub-Saharan Africa (SSA) on the antimicrobial activities of plants used to treat campylobacteriosis. The screened NPs may be candidates for in-vivo studies using different models.

The *C. jejuni* isolated from chicken (MT947450, CJ–CI) showed 100% similarity with *C. jejuni* (CP047481.1) isolated from patients with gastroenteritis in Chile. This shows the possible transmission of *Campylobacter* species from poultry to humans, and several reports have shown an association between human and poultry isolates when drinking contaminated water or eating undercooked meat [67,68]. Chicken is known as the major reservoir of human campylobacteriosis due to its high body temperature, which is suitable for *Campylobacter* growth [69], and increased poultry consumption [69,70]. Therefore, control measures and adherence to hygienic practices are required to reduce the transmission of *Campylobacter* from animals to humans. We also recommend studies on the synergistic activities of both NPs and existing antibiotics aimed at reducing the MIC values of drugs of choice and, thus, helping to slow down antimicrobial resistance and extend the effectiveness of existing antibiotics.

## 4. Materials and Methods 

### 4.1. Sampling Site

Chicken fecal samples were collected from a layer poultry farm located in Gangneung city, Republic of Korea. The farm uses an intensive poultry rearing system, and chickens are dispatched into battery cages inside a closed house. The farm adheres to hygienic practices by the use of footbath disinfectant at the entrance and cleanliness inside the farm.

### 4.2. Sample Collection, Campylobacter Isolation, and Antimicrobial Testing 

Pen floor fecal samples were collected using sterile cotton swabs, which were then placed on ice and transported to the laboratory within one hour. These samples were inoculated onto modified charcoal cefoperazone deoxycholate agar (mCCDA) (Oxoid Ltd., Basingstoke, UK) containing the *Campylobacter* mCCDA selective supplement, SR155E (Oxoid Ltd.). Plates were incubated at 37 °C for 48 h under microaerophilic conditions generated by CampyGen^TM^ gas sachets (Oxoid Ltd.), as previously described [71]. Typical colonies of *Campylobacter*, with the features of being moistened, gray, flat, and a tendency to spread [72], were subcultured on Mueller Hinton agar supplemented with 5% defibrinated horse blood and incubated at 37 °C for 48 h under microaerophilic conditions generated by CampyGen^TM^ gas sachets (Oxoid Ltd.). Species confirmation was performed by PCR and sequencing. *Campylobacter* isolates were preserved at −80 °C in Mueller Hinton broth (MHB) supplemented with 25% glycerol (*v*/*v*). Apart from chicken isolates, *Campylobacter jejuni* (ATCC^®^ 33560^TM^) and *Campylobacter coli* (ATCC^®^ 33559^TM^) were also used. For antibacterial activity assays, bacterial inoculum of 0.5 McFarland (1–5 × 10^8^ CFU/mL) was prepared from fresh colonies taken from MHA plates supplemented with 5% defibrinated horse blood (Oxoid Ltd., Basingstoke, Hampshire, England) and dissolved in sterile normal saline (0.85%). The absorbance was recorded spectrophotometrically at 600 nm (Synergy HT; BioTek Instruments Inc., Winooski, VT, USA).

Four strains were used in this study. In the case of *C. jejuni*, the reference strain (ATCC^®^ 33560^TM^) and the chicken isolate (MT947450) were named CJ–RS and CJ–CI, respectively. In the case of *C. coli*, the reference strain (ATCC^®^ 33559^TM^) and the chicken isolate (MT947451) were named CC–RS and CC–CI, respectively.

### 4.3. DNA Extraction, PCR, and Sequencing

Genomic DNA was extracted from pure colonies using the Qiagen QIAamp^®^ PowerFecal^®^ kit (Qiagen, Hilden, Germany), according to the manufacturer’s instructions, followed by multiplex-PCR using genus-specific primers (C412F; C1228R)*, cj0414* gene primers (C1; C3), and *ask* gene primers (CC18F; CC519R) (Table 3), as previously described with modification [73]. *cj0414* is a conserved gene coding for a fragment of a putative oxidoreductase subunit gene (PID 6967888; Cj0414) of *C. jejuni*, while *ask* encodes aspartokinase, highly specific for *C. coli* [74,75,76]. The PCR mixture (25 μL) contained 12.5 μL of 2× Master Mix (Thermo Fisher Scientific, Seoul, Korea), 1 μL of each primer, 1.5 μL of DNA, and 7 μL of sterile deionized water. The cycling conditions were one cycle of 95 °C for 5 min, 35 cycles each of 94 °C for 30 sec, 55 °C for 45 sec, and 72 °C for 45 sec, and a final extension at 72 °C for 7 min using a MiniAmp^TM^ Plus thermal cycler (Applied Biosystems, MA, USA). The PCR products were held at 4 °C before analysis. 

For the antibiotic resistance genes (*tet*(O), *gyr*A, and *cme*B), m-PCR was performed using specific primers (Table 3), as previously described [77,78]. PCR products were analyzed by gel electrophoresis. The bands of the amplification products were compared to the Dyne 100 bp DNA ladder (Dyne bio, Seongnam, Korea). Bands of PCR products were observed and photographed with an iBright™ CL1000 imaging system (Thermo Fisher Scientific, Seoul, Korea). The purification of PCR products was performed with the Pure Link^TM^ Quick PCR purification kit (Invitrogen, Vilnius, Lithuania) and sequenced by the Sanger method at SolGent (Solutions for Genetic technologies, Daejeon, Korea).

### 4.4. Natural Products and Antibiotics

#### 4.4.1. Plant Extracts, EOs, Pure Phytochemicals, and Conventional Antimicrobials

Plant extracts (Table 1), kuraridin and emodin, were obtained from the library of KIST Gangneung Institute of Natural Products. Except for *Cinnamomum* cassia (L.) J.Presl (BEA585A1), extracts were prepared by heat reflux extraction performed with a 10-g dried plant and 0.1 L ethanol for 2 h, twice. The ethyl acetate extract of BEA585A1 was obtained by fractionation with ethyl acetate from the water extract of dried bark of cinnamon, prepared with water reflux for 2 h. Essential oils (clove, cinnamon bark), pure phytochemicals (eugenol, trans (E)-Cinnamaldehyde, and baicalein), and antibiotics (ciprofloxacin, erythromycin, tetracycline, nalidixic acid, and gentamicin) were supplied from Sigma-Aldrich (St. Louis, MO, USA). Stock solutions of the plant extracts and EOs were dissolved in dimethylsulphoxide (DMSO). Antibiotics were dissolved as per the manufacturer’s instructions. Ciprofloxacin and erythromycin were dissolved in 0.1 N HCl and 70% ethanol, respectively. Gentamicin and tetracycline were dissolved in water, while nalidixic acid was dissolved in DMSO. The solutions of antibiotics were filter-sterilized before being used.

#### 4.4.2. Determination of MIC and MBC

The MIC and MBC for the tested NPs and antibiotics were determined by broth microdilution using 96-well plates (Greiner-bio-one, Kremsmünster, Austria) [79]. Briefly, 100 μL of the antimicrobials at working concentrations were pipetted to the first column of a plate. After two-fold serial dilutions by MHB across the plate, all wells were inoculated with 50 μL of inoculum except for the negative controls. Control wells were prepared with culture medium (sterility control), plant extract (negative control), bacterial suspension (positive control), and DMSO in amounts corresponding to the highest quantity present. The highest amount of DMSO in the test well was 2% for extracts and less than 0.5% for essential oils and pure phytochemicals. The DMSO at the highest concentration (2%) did not affect bacterial growth, as previously described [80]. Then, incubation was done at 37 °C for 48 h in microaerophilic conditions. All tests for antimicrobial susceptibility were repeated six times for reproducibility. The MICs were evaluated spectrophotometrically by measuring the bacterial concentration at an absorbance of 600 nm using a microplate reader (Synergy HT; BioTek Instruments Inc., Winooski, VT, USA). The MIC was confirmed by the addition of iodonitrotetrazolium chloride (INT), followed by agitation at 37 °C for 30 min in the dark. The MIC was defined as the lowest concentration of the antimicrobial agent that results in a significant decrease (˃90%) in inoculum viability after 48 h, as previously described, with modification on incubation time [81]. Bacterial growth was indicated by the presence of a pink color after the incubation period [82].

The MBC was determined as previously described, with modification [80]. From the wells which did not show growth, a volume of 10 μL was pipetted and streaked on the surface of MHA plates supplemented with 5% defibrinated horse blood (Oxoid Ltd., Basingstoke, Hampshire, England). The MBC was defined as the lowest concentration showing no growth after 48 h of incubation. The MIC values for antibiotics were assessed as per the epidemiological cut-off values of the European Committee for Antimicrobial Susceptibility Testing (EUCAST, http://www.eucast.org).

#### 4.4.3. Data Analysis

The MIC values were expressed as mean ± standard deviation (SD) for analysis performed in six replicates. One-way analysis of variance (ANOVA) was performed in GraphPad Prism 8.4.0 (GraphPad Software, La Jolla, CA, USA), and the differences among group means were verified by Tukey’s multiple comparisons test, with *p*-value < 0.05 considered as significant.

After the sequencing of PCR products, BioEdit software (version 7.2.6.1) was used to edit, align, and analyze the DNA sequences [83]. The consensus sequences obtained were compared to GenBank strains by a BLAST search, and they were submitted to GenBank to get accession numbers [84]. Standard sensitive strains for *gyr*A mutation, including *C. jejuni* (GenBank accession number L04566.1) and *C. coli* (GenBank accession number U63413.1), were used for comparison with the sequences of this study. For comparison, strains with *gyr*A mutations were also included (KX982339.1 and MT176401.1). For the *tet*(O) gene analysis, different GenBank accession numbers (AM884250, 81-176, and CP044175.1) were used. For the antibiotic resistance genes, sequence alignments were performed with Clustal Omega [85]. Amino acid sequences were deduced from the DNA sequences using the ExPASyTranslate tool [86].

## 5. Conclusions

The isolates from chicken were sensitive to erythromycin and gentamicin, but they were resistant to quinolones and tetracycline. The mutations in *gyr*A and *tet*(O) were confirmed by DNA sequencing. The tested NPs were active against both antibiotic-sensitive and antibiotic-resistant *Campylobacter* strains. Effective NPs can be exploited by the food processing industry and poultry farms to control foodborne pathogens. There is a need to understand the mode of action of these NPs before they are used in clinical settings. 

## Figures and Tables

**Table 1:** Information on used plant extracts.

Library Code	Family	Scientific Name	Common Name	Collection Site	Collection Date	Part of Plant	Extraction Solvent
BE0005B1	Lamiaceae	*Meehania urticifolia* (Miq.) Makino	Nettle-leaf mint	Gangneung, Gangwon	2016	Aerial part	Ethanol
BE0165A1	Lamiaceae	*Scutellaria baicalensis* Georgi	Skullcap	Yeosu, Jeonnam	2017	Root	Ethanol
BE0167A1	Lamiaceae	*Mentha canadensis* L.	Wild mint	Andong, Gyeongbuk	2017	Aerial part	Ethanol
BE1192A1	Lamiaceae	*Salvia plebeia* R.Br.	Common sage	Paju,	2015	Whole plant	Ethanol
				Gyeonggi			
BEA585A1	Lauraceae	*Cinnamomum* cassia (L.) J.Presl	Cinnamon	Gyeongdong Seoul	2015	Bark	Ethyl acetate

**Table 2:** The minimum inhibitory concentrations (MICs) and minimal bactericidal concentrations (MBCs) in µg/mL of different natural products (NPs) and antibiotics against *Campylobacter* strains.

NP/Antibiotic	CJ–RS		CC–RS		CJ–CI		CC–CI	
	MIC	MBC	MIC	MBC	MIC	MBC	MIC	MBC
*M. urticifolia*	400	800	400	800	400	800	400	800
*S. baicalensis*	400	800	400	800	400	800	400	800
*M. canadensis*	400	800	400	800	800	1600	400	800
*S. plebeia*	400	800	400	800	800	1600	400	800
*C. cassia*	200	400	200	400	200	400	200	400
Clove oil	50	100	100	400	50	100	200	400
Cinnamon oil	25	25	50	100	25	50	50	100
Eugenol	50	100	100	200	50	100	100	200
(E)-Cinnamaldehyde	25	25	50	50	25	50	50	50
Baicalein	32	64	64	64	32	64	64	64
Kuraridin	48	ND	48	ND	48	ND	48	ND
Emodin	50	ND	200	ND	50	ND	200	ND
Ciprofloxacin	0.125	1	0.5	1	32	64	64	128
Erythromycin	0.5	1	1	4	0.5	1	2	4
Gentamicin	2	8	2	8	1	2	1	8
Tetracycline	1	4	1	4	256	512	64	128
Nalidixic acid	16	32	8	32	128	256	64	128

ND = not determined.

**Table 3:** Target genes, primer sequences, and amplification conditions.

Target Gene	Primer Name	Sequence (5’–3’)	Amplicon Size	Annealing T (°C)	Reference
16S rRNA	C412F	GGATGACACTTTTCGGAGC	816	55	[73]
	C1228R	CATTGTAGCACGTGTGTC			
*cj0414*	C1F	CAAATAAAGTTAGAGGTAGAATGT	161		
	C3R	CCATAAGCACTAGCTAGCTGAT			
*ask*	CC18F	GGTATGATTTCTACAAAGCGAG	502		
	CC519R	ATAAAAGACTATCGTCGCGTG			
*tet*(O)	*tet*(O)F	GCGTTTTGTTTATGTGCG	559		[77,78]
	*tet*(O)R	ATGGACAACCCGACAGAAG			
*cjgyr*A	QRDRF	GCCTGACGCAAGAGATGGTTTA	454		
	QRDRR	TATGAGGCGGGATGTTTGTCG			
*cme*B	*cme*BF	TCCTAGCAGCACAATATG	241		
	*cme*BR	AGCTTCGATAGCTGCATC			
